# Stress-induced phosphoprotein-1 maintains the stability of JAK2 in cancer cells

**DOI:** 10.18632/oncotarget.10500

**Published:** 2016-07-08

**Authors:** Chia-Lung Tsai, Angel Chao, Shih-Ming Jung, Chi-Neu Tsai, Chiao-Yun Lin, Shun-Hua Chen, Shih-Che Sue, Tzu-Hao Wang, Hsin-Shih Wang, Chyong-Huey Lai

**Affiliations:** ^1^ Genomic Medicine Research Core Laboratory, Chang Gung Memorial Hospital, Taoyuan, Taiwan; ^2^ Department of Obstetrics and Gynecology, Chang Gung Memorial Hospital and Chang Gung University, Taoyuan, Taiwan; ^3^ Department of Pathology, Chang Gung Memorial Hospital and Chang Gung University, Taoyuan, Taiwan; ^4^ Graduate Institute of Clinical Medical Sciences, Chang Gung University, Taoyuan, Taiwan; ^5^ Graduate Institute of Biomedical Science, School of Medicine, Chang Gung University, Taoyuan, Taiwan; ^6^ Department of Life Sciences, Institute of Bioinformatics and Structural Biology, National Tsing Hua University, Taiwan; ^7^ School of Traditional Chinese Medicine, College of Medicine, Chang Gung University, Taoyuan, Taiwan

**Keywords:** JAK2, STIP1, cancer

## Abstract

Overexpression of stress-induced phosphoprotein 1 (STIP1) − a co-chaperone of heat shock protein (HSP) 70/HSP90 – and activation of the JAK2-STAT3 pathway occur in several tumors. Combined treatment with a HSP90 inhibitor and a JAK2 inhibitor exert synergistic anti-cancer effects. Here, we show that STIP1 stabilizes JAK2 protein in ovarian and endometrial cancer cells. Knock-down of endogenous STIP1 decreased JAK2 and phospho-STAT3 protein levels. The N-terminal fragment of STIP1 interacts with the N-terminus of JAK2, whereas the C-terminal DP2 domain of STIP1 mediates the interaction with HSP90 and STAT3. A peptide fragment in the DP2 domain of STIP1 (peptide 520) disrupted the interaction between STIP1 and HSP90 and induced cell death through JAK2 suppression. In an animal model, treatment with peptide 520 inhibited tumor growth. In summary, STIP1 modulates the function of the HSP90-JAK2-STAT3 complex. Peptide 520 may have therapeutic potential in the treatment of JAK2-overexpressing tumors.

## INTRODUCTION

Stress-induced phosphoprotein 1 (STIP1; Gene ID:10963) − a 62.6-kDa protein also known as heat shock protein (HSP)-organizing protein [1] – was initially identified in yeast [2]. All of the STIP1 homologues across species contain nine tetratricopeptide repeat (TPR) motifs that are clustered into three TPR (TPR1, TPR2A, and TPR2B) domains and two DP domains (DP1 and DP2) rich in aspartate and proline [3]. The TPR1 and TPR2B domains interact with HSP70, whereas the TPR2A and TPR2B domains are involved in the binding of STIP1 to HSP90 [4-6]. TPR2A recognizes the C-terminal pentapeptide (MEEVD) of HSP90 [4, 7]. The DP domains are α-helical folds that form a groove capable of binding to other proteins [5].

STIP1 functions as an adapter that directs HSP90 to HSP70-client protein complexes in the cytoplasm, ultimately modulating their chaperone activity [1]. These complexes are involved in a number of different cellular activities, including RNA splicing, transcription, protein folding, signal transduction, and cell cycle regulation [8, 9]. Studies in mice have shown that complete STIP1 loss-of-function causes embryonic lethality, increased caspase 3 activation, impaired cell proliferation and reduced expression of several HSP90 client proteins [10]. However, the exact functions of DP1 and DP2 domains have not been entirely elucidated. *In vivo* experiments demonstrated that the TPR2A-TPR2B-DP2 module is involved in client activation [5]. Moreover, mutational analyses revealed that the DP2 domain is critical when the glucocorticoid receptor is activated [5].

Several malignancies including hepatocellular carcinoma [11], pancreatic cancer [12], ovarian cancer [13, 14], colon cancer [15], and cholangiocellular carcinoma [16] are characterized by STIP1 overexpression. In cancer cells, knockdown of STIP1 expression has been shown to reduce tumor invasiveness through the downregulation of matrix metalloproteinase-2 [17] and RhoC GTPase and related inhibition of pseudopodia formation [18]. In addition, STIP1 knock-down decreased the expression of HSP90 client proteins (e.g., HER2, Bcr-Abl, c-MET, and v-Src) [17]. Interestingly, clinical studies demonstrated that an increased STIP1 protein expression portends adverse outcomes in ovarian cancer [13]. STIP1 may also serve as a potential biomarker for cholangiocellular carcinoma [16] and hepatocellular carcinoma [11].

Knock-down of STIP1 has been shown to suppress signal transducer and activator of transcription 3 (STAT3) mRNA levels in mouse embryonic stem cells, inhibiting their pluripotent capacity to form embryoid bodies [19]. STAT3 is involved in the interleukin (IL)-6-type cytokine signaling that plays a key role in normal cell function as well as in a number of different disease conditions [20]. In this regard, activation of the IL6-Janus kinase 2 (JAK2)-STAT3 pathway has been observed in myeloproliferative disorders [21] and ovarian cancer [22]. STAT3 phosphorylation promotes its dimerization and translocation into the nucleus to function as a transcriptional modulator, playing an important role in the regulation of cell proliferation, apoptosis, and angiogenesis [22].

Activation of the IL6-JAK2-STAT3 pathway is also regulated by the HSP90 chaperone machinery [23]. The N-terminal domain of HSP90 can directly bind the SH2 DNA binding domain of STAT3 [24]. JAK2 may be degraded through the use of HSP90 inhibitors in human leukemic cells [25]. Furthermore, HSP90 inhibitors can abrogate JAK inhibitor resistance, suggesting the superiority of combined therapy with HSP90 and JAK inhibitors [26, 27].

In the current study, we demonstrate that STIP1 maintains the stability of JAK2 protein. Interestingly, both a HSP90 C-terminal inhibitor and a specific STIP1 peptide that blocks the STIP1-HSP90 interaction were able to suppress JAK2 protein expression. In addition, we identified the DP2 domain of STIP1 as an important regulator of the JAK2-STAT3 pathway. A peptide 520 derived from the DP2 domain of STIP1 was capable of suppressing JAK2 protein expression. In addition, it blocked STAT3 phosphorylation and induced cell death both *in vitro* and *in vivo*.

## RESULTS

### STIP1 maintains JAK2 protein stability

**Figure 1:**
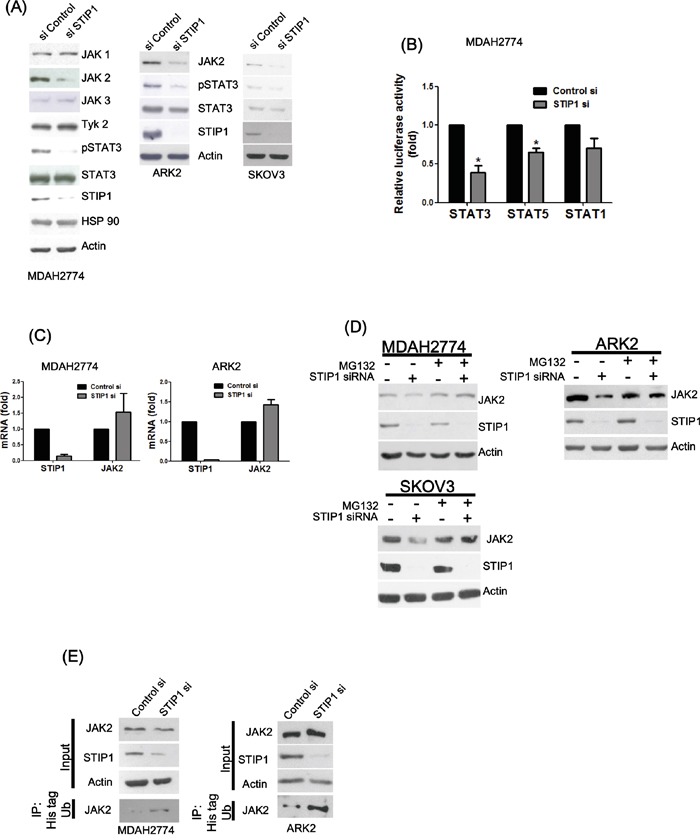
STIP1 maintains JAK2 protein stability in cancer cells **A.** Ovarian (MDAH2774 and SKOV3) and endometrial (ARK2) cancer cells were transfected with control or STIP1 siRNA. JAK2 and phospho-STAT3 protein levels were determined by western blot. Actin levels were used to normalize the input proteins. **B.** MDAH2774 cells were transfected using the mentioned STATs (GAS1/STAT1, STAT3, and STAT5) reporter constructs, transfected with control or STIP1 siRNA, and treated with IL-6 (50 ng/mL) for 24 h. The reporter activity was measured using the luciferase assay. Results are means ± standard errors from three independent experiments. Statistical significance was calculated with the Student's *t*-test, *P <0.05. **C.** MDAH2774 and ARK2 cells were transfected with control or STIP1 siRNA. STIP1 and JAK2 RNA levels were measured with real-time quantitative PCR at 72 h after siRNA transfection. Results are means ± standard errors from three independent experiments. GAPDH was used for normalization. **D.** Cancer cells (MDAH2774, ARK2, and SKOV3) were treated with MG132 (25 μM) for 6 h either in the presence or absence of STIP1 siRNA. JAK2 and STIP1 protein levels were determined by western blot. **E.** Cancer cells (ARK2 and MDAH2774) either with or without STIP1 knockdown were transfected with His-tagged ubiquitin (His-Ub). His-Ub-labeled were immunoprecipitated with nickel beads. JAK2 levels were determined by western blot. The same quantity of protein lysates (50 μg) was used as loading control and probed with anti-JAK2, anti-STIP1, and anti-actin antibodies.

Both STIP1 and STAT3 have been shown to form a heterocomplex in the HSP70/HSP90 chaperone machinery [19]. We therefore used RNA interference to examine the role of STIP1 in the regulation of the JAK-STAT pathway in ovarian and endometrial cancer cell lines. STIP1 knock-down significantly suppressed JAK2 and phospho-STAT3, but no significant effects on JAK1, JAK3, TYK2, and total STAT3 were observed (Figure 1A). STIP1 knock-down markedly inhibited the IL6-stimulated STAT3 transcriptional activity, whereas only a partial block was observed for STAT5. In contrast, STAT1 was not significantly suppressed (Figure 1B). Surprisingly, STIP1 knock-down did not decrease JAK2 mRNA; a slight increase was noted instead (Figure 1C). The amount of JAK2 protein increased in STIP1-knocked-down cancer cells after exposure to MG132 (Figure 1D). Furthermore, JAK2 ubiquitination was increased when cotransfection with histidine-tagged ubiquitin was performed in STIP1-knocked-down cells (Figure 1E). The observation that STIP1 knock-down increased JAK2 degradation suggests that STIP1 stabilizes the JAK2 protein by inhibiting proteasome-mediated degradation.

### STIP1 is involved in the formation of the JAK2-HSP90-STAT3 complex

**Figure 2:**
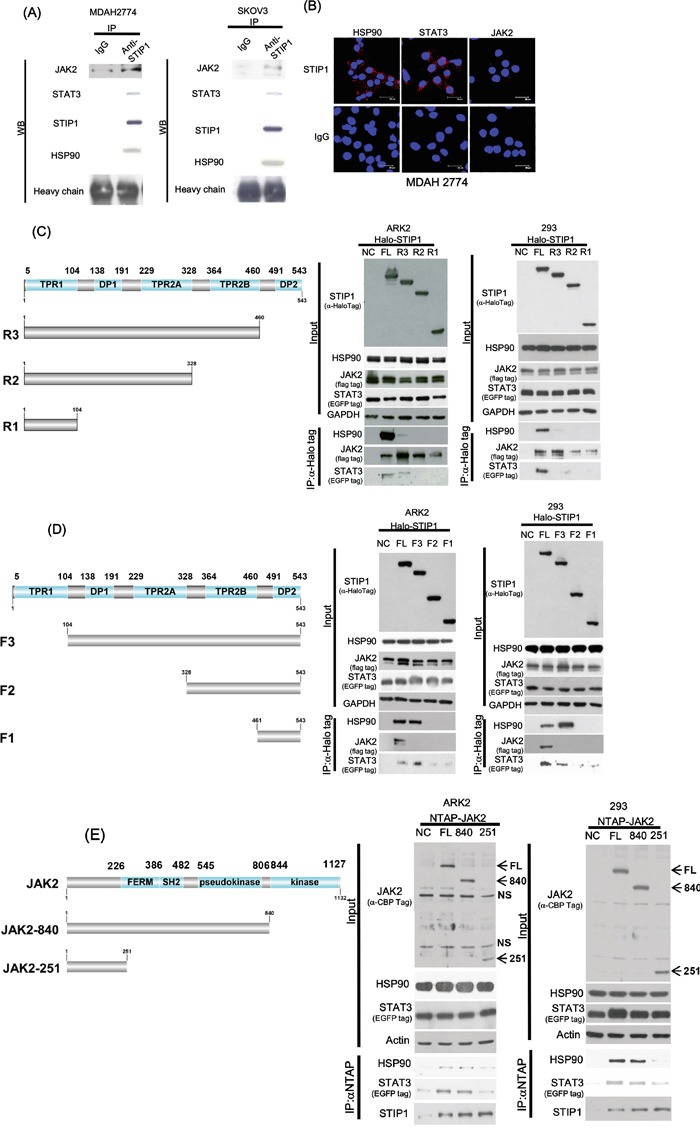
STIP1 is essential for the formation of the JAK2-HSP90-STAT3 complex **A.** Endogenous STIP1 protein complexes obtained from protein lysates (2 mg) of ovarian cancer cells (MDAH2774 and SKOV3) were immunoprecipitated with anti-STIP1 or control IgG antibodies. The protein associations of halo-STIP1 with HSP90, JAK2, or STAT3 were determined with western blot. **B.** MDAH2774 cells were grown on coverslips. Cells were permeabilized, and an *in situ* proximity ligation assay (PLA) was performed to investigate protein interactions (red dots). To this aim, anti-STIP1 and anti-HSP90 (left-upper panel), anti-STIP1 and anti-STAT3 (middle-upper panel), and anti-STIP1 and anti-JAK2 (right-upper panel) antibodies were used. An IgG was used as a negative control in place of the anti-STIP1 antibody (lower panel). **C, D.** The truncated STIP1 constructs used for the study are shown in left panels. They included the C-terminal truncated halo-tagged STIP1s (FL: full length, R3: DP2 deleted, R2: TPR2B-DP2 deleted, and R1: DP1-TPR2A-TPR2B-DP2 deleted) (C) and the N-terminal truncated halo-tagged STIP1s (FL: full length, F3: TPR1 deleted, F2: TPR1-DP1-TPR2A deleted and F1: TPR1-DP1-TPR2A-TPR2B deleted) (D). ARK2 and 293 cells were co-transfected with the reported truncated constructs of STIP1, Flag-JAK2, and EGFP-STAT3, and subsequently purified with Halo-tag resin. Co-immunoprecipitated HSP90, JAK2, and STAT3 were analyzed with western blot using anti-HSP90, anti-Flag, and anti-EGFP antibodies, respectively. **E.** ARK2 and 293 cells were co-transfected with full-length NTAP-JAK2 or its truncated constructs (JAK2-840, JAK2-521) and EGFP-STAT3. Co-immunoprecipitated HSP90, STIP1, and STAT3 were analyzed with western blot using specific antibodies. NTAP-JAK2 constructs were detected using an anti-calmodulin binding peptide (CBP) antibody. NS denotes non-specific band detected with the CBP antibody. FL: full-length JAK2, 840: protein of the JAK2-840 construct, 251: protein of the JAK2-251 construct.

To investigate whether STIP1, JAK2, STAT3, and HSP90 may form a complex in cancer cells, STIP1 was pulled-down using a specific antibody. JAK2, STIP1, and HSP90 were all detectable in STIP1-pulled down complexes (Figure 2A). In proximity ligation assay (PLA), two primary antibodies are used to target the protein in close proximity, followed by the use of secondary antibodies with a unique short DNA strand. When two primary antibodies are in close proximity, the DNA strands of secondary antibodies can be amplified by DNA polymerase and labeled with fluorescent complementary probes. Each of the STIP1-HSP90, STIP1-STAT3, and STIP1-JAK2 interactions was confirmed using the PLA (Figure 2B).

Systematically truncated constructs of STIP1 and JAK2 revealed a number of interactions (Figure 2C-2E). Deletion of DP2 in R3/STIP1 (Figure 2C) dramatically decreased its binding to HSP90, whereas deletion of TPR2B in R2/STIP1 (Figure 2C) or deletion of TPR2A in F2/STIP1 (Figure 2D) completely abrogated the HSP90-STIP1 interaction. These results indicate that both TPR2A and TPR2B are necessary for STIP1 interaction with HSP90 and that DP2 is important for stabilizing the interaction between STIP1 and HSP90. However, none of TPR2A, TPR2B, DP2 alone, or only TPR2B-DP2, is sufficient for STIP1 binding to HSP90.

Deletion of TPR2A in F2/STIP1 and deletion of TPR2B in F1/STIP1 (Figure 2D) decreased the interaction between STIP1 and STAT3, but the absence of DP2 in R1/STIP1, R2/STIP1, and R3/STIP1 completely abolished its binding to STAT3 (Figure 2C). Deletion of TPR1 in F1/STIP1, F2/STIP1, and F3/STIP1 resulted in a complete absence of the STIP1-JAK2 interaction (Figure 2D). In contrast, the N-terminal segment (251 amino acids) of JAK2 ensured a sufficient binding to STIP1 (Figure 2E). Notably, the central portion of JAK2 (from amino acid 252 to amino acid 840) was necessary for the interaction between HSP90 and STAT3 (Figure 2E).

Taken together, these results suggest that STIP1 forms a complex with JAK2, STAT3, and HSP90. JAK2 binds to the N-terminal TPR1 domain of STIP1, whereas HSP90 and STAT3 interact with the C-terminal TPR2A-TPR2B-DP2 domains of STIP1. The N-terminal region of JAK2 is involved in the interaction with STIP1, whereas the JAK2 central region interacts with HSP90 and STAT3.

### The interaction between STIP1 and HSP90 is important to maintain JAK2 protein stability

**Figure 3:**
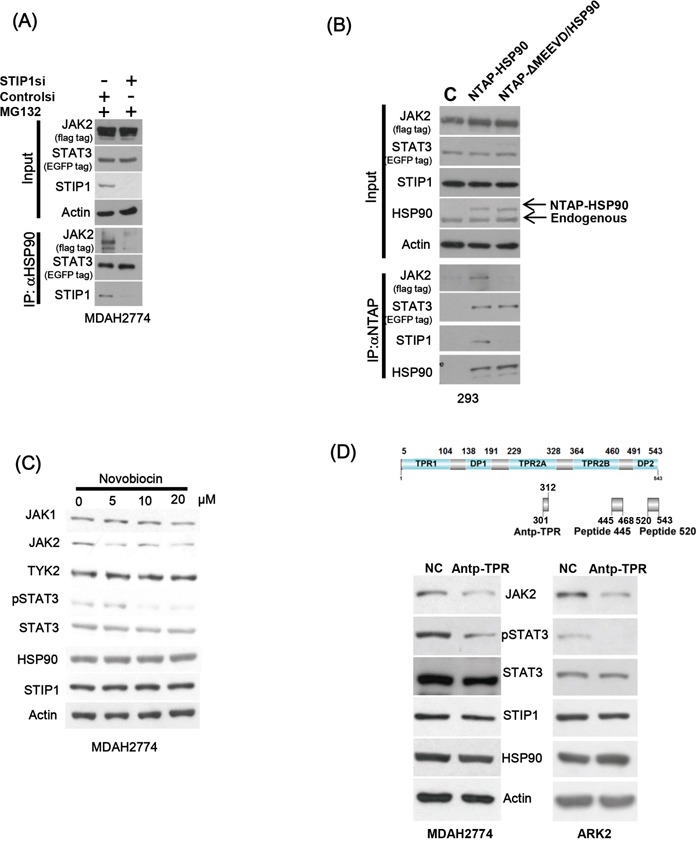
The interaction between STIP1 and HSP90 plays an important role in JAK2 stability **A.** MDAH2774 cells were co-transfected with EGFP-STAT3 and Flag-JAK2 expression vectors; STIP1 expression was subsequently knocked down with STIP1 siRNA. Resultant cells were treated with MG132 and immunoprecipitated with anti-HSP90. The proteins involved in the formation of HSP90 complexes (STIP1, JAK2, and STAT3) were identified with western blot using specific antibodies. **B.** 293 cells were co-transfected with NTAP-HSP90 or the corresponding form bearing a deletion of five amino acids (720−724) in the C-terminus (NTAP-ΔMEEVD/HSP90), Flag-JAK2, and EGFP-STAT3. Pulled-down proteins were analyzed with specific antibodies. **C.** MDAH2774 cells were treated for 24 h with different concentrations of Novobiocin, a HSP90 C-terminal inhibitor. Protein levels of JAK2, HSP90, STIP1, and phospho-STAT3 were determined with western blot. **D.** The upper panel shows the positions of Antp-TPR peptide, peptide 445, and peptide 520 in STIP1. MDAH2774 cells were transiently transfected with peptide 445 or peptide 520 for 24 or 48 h. They were subsequently analyzed with western blot using the reported antibodies.

Knock-down of endogenous STIP1 alone inhibited HSP90 and JAK2 binding, but no effects were observed on the HSP90-STAT3 interaction (Figure 3A). In contrast, a truncated form of HSP90 lacking five amino acids in the C-terminus (ΔMEEVD) showed a decreased interaction with both JAK2 and STIP1 (Figure 3B). Novobiocin is an antibiotic that binds to the HSP90 C-terminal domain [28] and is capable of suppressing the interaction of HSP90 with its co-chaperones (including STIP1) [29]. Protein levels of JAK2 and phospho-STAT3 decreased when cells were exposed to increasing novobiocin concentrations, although no effects on other JAK kinases and total STAT3 were observed (Figure 3C). To shed more light on the potential importance of the STIP1-HSP90 interaction for JAK2 stability, cancer cells were treated with the Antp-TPR peptide derived from the TPR2A domain of STIP1 [30]. Notably, levels of JAK2 and phospho-STAT3 were dramatically suppressed by Antp-TPR peptide treatment (Figure 3D).

### Peptide 520 blocks the STIP1-HSP90 interaction and suppresses JAK2 expression

**Figure 4:**
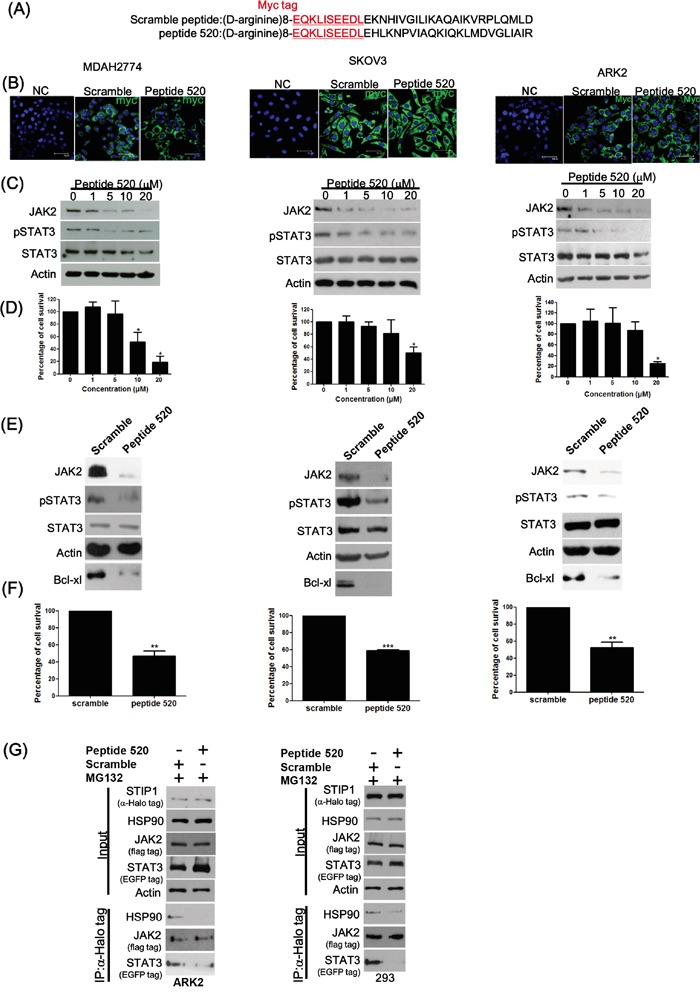
Peptide 520 blocks the JAK2-STAT3-Bcl X_L_ pathway and induces cell death in cancer cells **A.** Peptide 520 was synthesized with eight D-arginine followed by the myc-tag sequence in the N-terminus. The scramble peptide was synthesized using the same 24 amino acids of the peptide 520 in random order. **B.** MDAH2774 (left panel), SKOV3 (middle panel), and ARK2 (right panel) cells were transfected with peptide 520 and the sramble peptide. They were subsequently analyzed with immunofluorescence microscopy using an anti-myc antibody. **C, D.** MDAH2774 (left panel), SKOV3 (middle panel), and ARK2 (right panel) cells were treated with different concentrations of peptide 520. JAK2 and phospho-STAT3 protein levels were determined with western blot (C). Cell survival was investigated with the MTT assay (D). **E, F.** MDAH2774 (left panel), SKOV3 (middle panel), and ARK2 (right panel) cells were transfected with either peptide 520 or scramble peptide (20 μM). Protein levels of JAK2, phospho-STAT3, and Bcl X_L_ were determined with western blot (E). Cell survival was investigated with the MTT assay (F). **G.** Compared with exposure to the scramble peptide (10 μM) for 24 h, peptide 520 interfered with the formation of the JAK2-STIP1-HSP90-STAT3 complex.

We have previously shown that anti-STIP1 antibodies can inhibit malignant cell proliferation [6]. Here, we used antibody transfection experiments with three STIP1 monoclonal antibodies (M02, M04, and M06) to investigate whether endogenous STIP1 is involved in cancer cell survival (Supplementary Figure S1A). Cell cytotoxicity and apoptosis were more pronounced when cells were transfected with the M02 antibody (Supplementary Figure S1B and S1C). We synthesized seven peptides tiling over amino acids 445−543 of STIP1 to map the M02 antibody recognition site. Of the tested peptides, peptide 520 (corresponding to amino acids 520−543 of STIP1) was the most robustly recognized by the M02 antibody (Supplementary Figure S1D). Peptide 520 at 1:6 molar ratio neutralized the recognition of recombinant human STIP1 (rhSTIP1) by the M02 antibody (Supplementary Figure S1E), suggesting that this amino acid stretch is the primary epitope for the M02 antibody. Surprisingly, transfection of peptide 520 alone induced cell death (Supplementary Figure S1F). We also observed that cells treated with peptide 520, but not with peptide 445, were characterized by inhibition of JAK2 protein (Supplementary Figure S1G). To investigate the mechanisms by which peptide 520 can block JAK2 expression, eight D-arginine residues and a myc-tag were added to the N-terminus of both peptide 520 and control peptide (Figure 4A). Cancer cells were exposed to peptide 520 (1 μM) for 24 h. Thereafter, peptides were clearly identifiable in the cytosol of treated cells (Figure 4B). Importantly, treatment with peptide 520 suppressed JAK2, phospho-STAT3 protein expression (Figure 4C), and induced cancer cell death (Figure 4D) in a dose-dependent manner.

The JAK2-STAT3 pathway is a transcriptional regulator involved in a number of cellular processes, including apoptosis. Bcl-xL is one of the anti-apoptotic proteins regulated by STAT3 in cancer cells, and its expression can be suppressed using specific inhibitors of the STAT3 pathway [31]. Treatment with peptide 520 suppressed JAK2, phospho-STAT3, and Bcl-xL (Figure 4E), resulting in cancer cell death (Figure 4F). Peptide 520 was also able to reduce JAK2 and Bcl-xL protein levels in HEL cells harboring the JAK2 V617F mutation (Supplementary Figure S2). The effects of peptide 520 on disrupting the interactions in STIP1-HSP90 and STIP1-STAT3 are specific (Figure 4G).

The C-terminal TPR2B and DP2 domains of STIP1 are necessary for HSP90 binding (shown by R2 in Figure 2C), and DP2 alone is important for stabilizing the interaction between STIP1 and HSP90 (shown by R3 in Figure 2). We therefore hypothesized that peptide 520 may interfere with the STIP1-HSP90 interaction. Treatment with peptide 520 suppressed JAK2 protein concentrations in a dose-dependent fashion, without affecting HSP90 and STAT3 protein levels. Treatment with peptide 520 reduced the interactions between STIP1 and HSP90, STIP1 and JAK2, as well as between STIP1 and STAT3 (Supplementary Figure S3A). Treatment with the proteasome inhibitor MG132 did not have a significant effect on the disrupted STIP1-HSP90 and STIP1-STAT3 interactions elicited by peptide 520 (Supplementary Figure S3A and S3B). However, MG132 was able to preserve JAK2 protein levels pulled-down by STIP1 when cells were treated with increasing doses of peptide 520 (Figure 4G and Supplementary Figure S3B). These results indicate that peptide 520 can affect STIP1-HSP90 interaction and reduce JAK2 protein levels through a protein degradation mechanism. Peptide 520 also interferes with the STIP1-STAT3 interaction. Taken together, our findings demonstrate that peptide 520 effectively blocks the JAK2-STAT3 pathway and acts as an inhibitor of its downstream anti-apoptotic protein Bcl-xL, ultimately causing cell death.

### STIP1 is positively correlated and interacts with JAK2 *in vivo*

**Figure 5:**
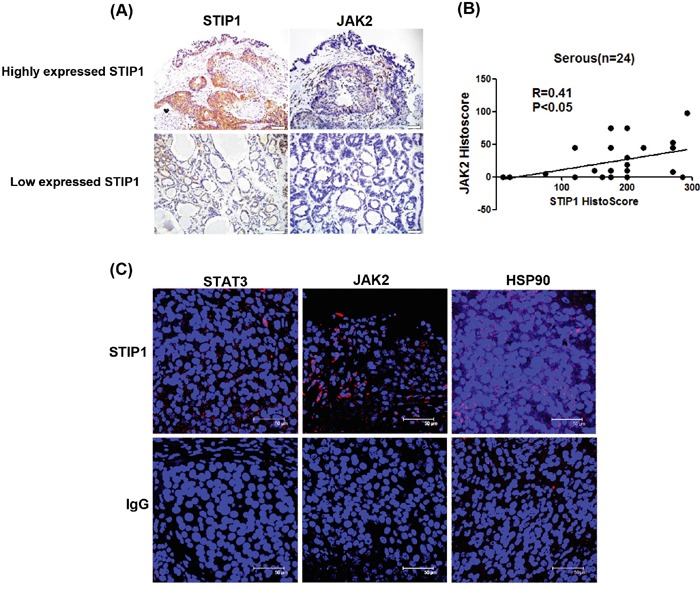
STIP1 and JAK2 are co-expressed in ovarian cancer tissues **A.** STIP1 and JAK2 protein levels in representative ovarian cancer tissues were determined by immunohistochemistry. Tumors with high and low STIP1 expression are shown in the upper and lower panels, respectively. The scale bar represents 100 μm. **B.** STIP1 and JAK2 immunostaining was analyzed with a histoscore. The correlation between STIP1 and JAK2 histoscores in ovarian serous carcinomas (n = 24) is reported. **C.** A proximity ligation assay (PLA) was performed to investigate protein interactions in representative ovarian cancer tissues. To this aim, anti-STIP1 and anti-STAT3 (left-upper panel), anti-STIP1 and anti-JAK2 (middle-upper panel), and anti-STIP1 and anti-HSP90 (right-upper panel) antibodies were used. An IgG was used as a negative control in place of the first antibody (lower panel).

Ovarian cancer tissues were subjected to immunohistochemistry (IHC) and PLA to investigate the *in vivo* interactions between STIP1 and JAK2. A high immunohistochemical expression of JAK2 was accompanied by an increased STIP1 expression (Figure 5A). Significantly positive correlations between STIP1 and JAK2 expression in serous ovarian cancers were identified (P < 0.05, Figure 5B). The positive correlations between STIP1 and JAK2 expression were also seen in other cancer types, including hepatocellular carcinoma and breast cancer (Supplementary Figure 4). PLA performed in formalin-fixed paraffin-embedded ovarian cancer tissues clearly demonstrated the interactions between STIP1 and STAT3, STIP1 and JAK2, as well as STIP1 and HSP90 localized mainly in cytoplasm (Figure 5C). Taken together, these results support the hypothesis that the HSP90-STIP1 complex maintains JAK2 stability and serve as a scaffold complex for the JAK2-STAT3 signaling pathway.

### Peptide 520 inhibits *in vivo* tumor growth

**Figure 6:**
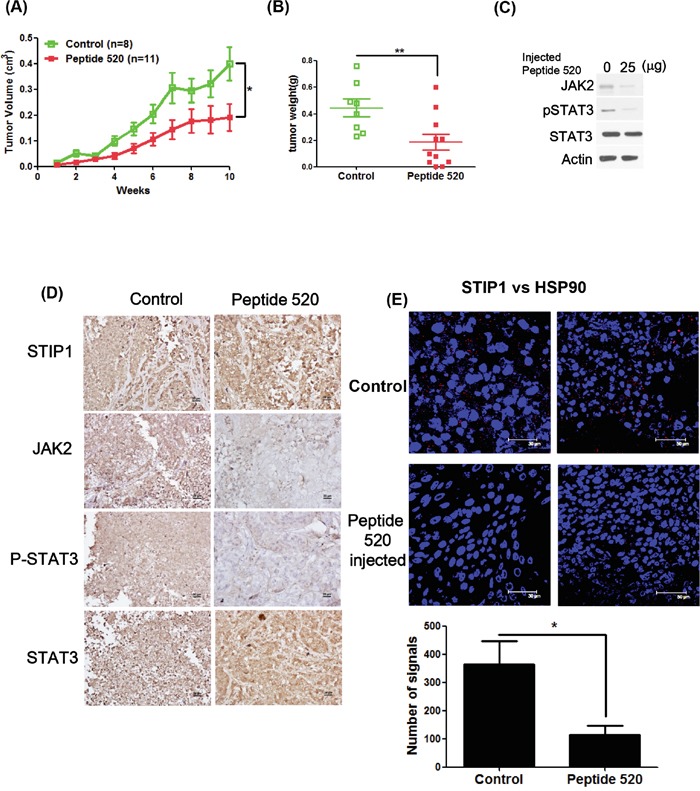
Peptide 520 blocks the JAK2-STAT3 pathway and inhibits tumor growth in mice **A, B.** MDAH2774 cells (1 × 10^6^) were subcutaneously injected into nude mice. When a tumor volume of 10 mm^3^ was reached, mice were injected with 50 μL PBS (vehicle alone) or 25 μg/50 μL peptide 520 directly into the tumor (three times per week). Tumor growth was measured on a weekly basis after treatment was started. Data on volume (A) and weight (B) are expressed as means ± standard. *P <0.05 and ** P <0.01, Student's *t*-test. **C, D.** Whole cell lysates from individual tumors were obtained from experimental mice. The JAK2, phospho-STAT3, and total STAT3 protein levels were determined with western blot (C) and immunohistochemistry (D). The scale bar represents 20 μm. **E.** The interactions between STIP1 and HSP90 were analyzed in representative tumors in nude mice (control group and peptide 520-treated group) with the proximity ligation assay. The left panel displays a higher magnification of the right panel. The signals were counted with Image J (https://imagej.nih.gov/ij/) (lower panel).

In a nude mouse model, treatment with peptide 520 inhibited ovarian cancer tumor growth (Figure 6A and 6B). Both western blot and IHC indicated that peptide 520 was able to inhibit JAK2 and phospho-STAT3 expression (Figure 6C and 6D). Furthermore, the *in vivo* interaction between STIP1 and HSP90 was inhibited by peptide 520 (Figure 6E). These results indicate that peptide 520 derived from the STIP1 DP2 domain may be useful for antagonizing JAK2/STAT3 activation in cancer cells.

## DISCUSSION

**Figure 7:**
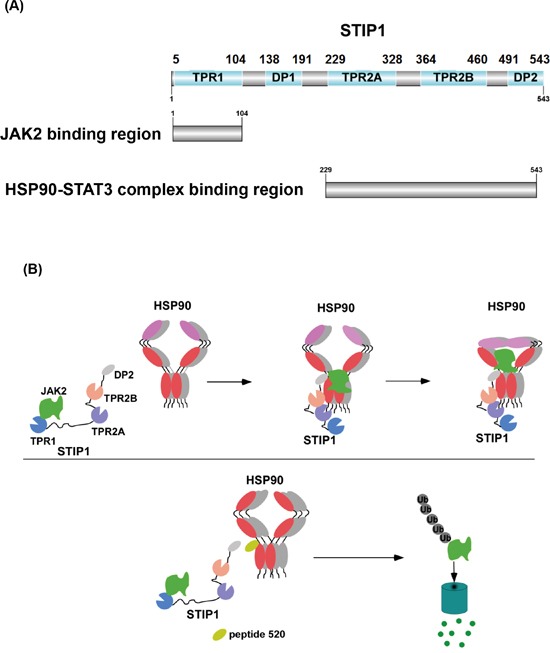
Model showing how the HSP90-STIP1 complex controls JAK2 kinase stability **A.** Summary of STIP1 domains that interact with JAK2, HSP90, and STAT3. **B.** STIP1 directly interacts with JAK2 and favors its delivery to HSP90 for proper protein folding, ultimately inhibiting its ubiquitin-dependent degradation in the proteasome (upper panel). Peptide 520 of the STIP1 DP2 domain blocks the interaction between STIP1 and HSP90, ultimately promoting JAK2 degradation (lower panel). Shadows of HSP90 indicated that molar ratios of these proteins were not determined in this study.

The results of our study indicate that HSP90 and its co-chaperone STIP1 are important regulators of the JAK2-STAT3 pathway. We also show for the first time that the N-terminus of STIP1 interacts with the N-terminus of JAK2 (Figure 2). Notably, JAK2 was unable to form a complex with HSP90 and STAT3 in the absence of STIP1 (Figure 2E). We therefore hypothesize that JAK2 initially forms a complex with STIP1, which is subsequently transported to the HSP90-STAT3 complex. The HSP90 chaperone machinery promotes appropriate folding of JAK2 and serves as scaffold protein for the JAK2-STAT3 signaling. When a peptide interfering with the HSP90-STIP1 interaction blocks the STIP1-mediated loading of JAK2 to the HSP90 chaperone machinery, JAK2 is degraded through ubiquitin pathway (Figure 7). These results suggest that 1) JAK2 is a client protein folded by the STIP1-HSP90 complex, and 2) a reduced expression endogenous STIP1 elicited by siRNA or the truncated C-terminal of HSP90 blocks JAK2-HSP90 interaction, ultimately reducing JAK2 and phospho-STAT3 levels.

Although the DP2 domain of STIP1 is known to play an important role in the activation of client proteins [5], the exact mechanisms remain to be elucidated. Nuclear magnetic resonance spectroscopy demonstrated that DP2 does not interact directly with purified HSP90 and HSP70, but it makes contacts with the TPR2B domain of STIP1 [5, 32]. DP2 domain forms a rigid C-terminal module with TPR2A and TPR2B domains [5, 32]. Pull-down experiments with isotopically-labeled STIP1 and rabbit reticulocyte lysate have shown that the ability to bind HSP70 is reduced when STIP1 has a truncated or mutant DP2 domain [33-35]. Here, we demonstrate that DP2-deleted STIP1 dramatically decrease its binding to HSP90 (Figure 2c), whereas peptide 520 can block the interaction between HSP90 and STIP1 both *in vitro* (Figure 4G and Supplementary Figure S3) and *in vivo* (Figure 6E). These results further corroborate the hypothesis that DP2 is required for loading client proteins to HSP90 and provide a potential explanation for the involvement of the DP2 domain in client protein activities [5]. Although TPR2A domain binds to MEEVD domain of HSP90, our results demonstrated that both TPR2A and DP2 are important for HSP90 binding, STIP1 cannot bind to HSP90 if either one domain is not functioning. Given that DP2 forms a rigid module with TPR2A-TPR2B, DP2 domain is important in client protein activation and plays a role in the interaction between HSP90 and the TPR2A-TRP2B-DP2 structure [32]. The complexes of clients with DP2-truncated STIP1 were unable to bind the HSP90 chaperone machinery. In turn, the incorrect folding caused degradation of client proteins.

HSP90 is an ATP-dependent molecular chaperone that operates with HSP70 and different co-chaperones in the maturation of client proteins. The block of the HSP90 chaperone machinery results in a degradation of client proteins via the ubiquitin-proteasome pathway. Because some client proteins (e.g., HER2, BRAF, and MET) play an important role in tumorigenesis, HSP90 is considered as a druggable target for cancer treatment. Most HSP90 inhibitors are designed to target the ATP-binding pocket in the N-terminus, ultimately competing with ATP binding [36]. An alternative approach consists in the use of small molecule inhibitors that disrupt protein-protein interactions in the HSP90 chaperone machinery [37]. Recent evidence indicates that small compounds or peptides that interfere with the interaction between the TPR2A domain of STIP1 and the MEEVD motif of HSP90 can display anti-cancer activity both *in vitro* and *in vivo* [38]. These results suggest the potential usefulness of alternative strategies in the development of novel HSP90 inhibitors. In line with this possibility, the results of our study indicate that peptide 520 can disrupt the interaction between HSP90 and STIP1 (Figure 4G and Supplementary Figure S3), ultimately reducing JAK2 protein levels and causing cell death through the suppression of the JAK2-STAT3 downstream anti-apoptotic protein Bcl-XL (Figure 4E and 4F).

HSP90 inhibitors may promote the degradation of JAK2 protein and overcome resistance to JAK2 inhibitors in JAK2 mutant cells [25, 27, 39]. JAK2 is associated with HSP90 as an HSP90 client, and HSP90 inhibitors may suppress JAK2 protein expression in both wild-type and JAK2 inhibitor-resistant mutant cancer cells [25]. The combined use of HSP90 inhibitors and JAK2 inhibitors has been proposed for diseases characterized by JAK2-STAT3 hyperactivation [26]. However, most JAK2 inhibitors are ATP-competitive compounds inhibiting JAK2 activity [40]. These inhibitors may develop drug resistance via novel mutations in JAK2 or off-target effects [40]. Peptide 520 does not directly target JAK2, thus its use may not result in these problems. In this study, we have shown that the formation of the JAK2-HSP90 complex occurs in a sequential manner. We propose the existence of two subcomplexes in the HSP90 chaperone machinery of the JAK2-STAT3 pathway, namely JAK2-STIP1 and HSP90-STAT3. The final functional complex is formed through the interaction of STIP1 with HSP90 (Figure 7).

We conclude that STIP1 overexpression in cancer cells is involved in the regulation of the JAK2-STAT3 pathway. JAK2, STIP1, and HSP90 form a complex required for both the maturation of the JAK2 protein and as a scaffold complex for the transduction of JAK2-STAT3 signaling. Repression of STIP1 or interruption of the STIP1-HSP90 interaction warrant further scrutiny as potential strategies for cancer treatment.

## MATERIALS AND METHODS

### Ethics statement

This study protocol complied with the tenets of the Helsinki declaration and was approved by the Institutional Review Board of the Chang Gung Memorial Hospital (CGMH-IRB #101-0372B).

### Culture and treatment of cell lines

Human ovarian cancer cell lines (SKOV3 and MDAH2774) and human embryonic kidney epithelial cells 293 were obtained from the American Type Culture Collection (Manassas, VA, USA). The human endometrial cancer cell line ARK2 was kindly provided by Dr. Alessandro D. Santin (Yale University, School of Medicine, New Haven, CT, USA). JAK2 V617F mutant cell-HEL cell [41] was a gift from Dr. Chao Tsu-Yi (Tri-Service General Hospital, National Defense Medical Center, Taipei, Taiwan) [42]. SKOV3, MDAH2774 and 293 cells were cultured in DEME/F12, whereas ARK2 and HEL cell were maintained in RPMI medium with 10% fetal bovine serum, 50 U/ml penicillin and 50μg/ml streptomycin. Before harvesting the cells in designated experiments, cells were pre-treated with 25 μM MG132 (proteasome inhibitor, Sigma-Aldrich, St. Louis, MO, USA) for 5 h, IL-6 50 ng/mL (JAK2-STAT3 activator, Millipore, Billerica, MA, USA) or novobiocin (HSP90 C-terminal inhibitor, Sigma-Aldrich) for 24 h.

### DNA constructs

The expression vector HaloTag STIP1 was purchased from Promega (Madison, WI, USA). Flag-JAK2 was kindly provided by Dr. Ludger Hengst (Innsbruck Medical University, Innsbruck, Austria) [43]. EGFP-STAT3 was a gift form Dr. Pravin Sehgal (New York Medical College, Valhalla, NY, USA) [44]. The HaloTag STIP1 deletion constructs were amplified from the HaloTag STIP1 (Kazusa DNA Research Institute, Chiba, Japan) expression vector with the forward primer 5′-GCGATCGCCATGGAGTCCGGCAGCCC-3′ and the following reverse primers R1: 5′-GTTTAAACTCAATTTGCCTCGTGTTTTAAGCC-3′, R2: 5′-GTTTAAACTCATGCCAGAGACTTGTTATAGAAATG-3′, and R3 5′-GTTTAAACTCAGCTGGAGTCCAGGTCTAGCG-3′ for the STIP1 C-terminal deletion. The forward primers F1 5′-GCGATCGCCATGAACCCTCAACTGAAAGAGGGT, F2 5′-GCGATCGCCATGGAGCACCGAACCCCAGATG-3′, and F3 5′-GCGATCGCCATGTGTAAGGAGGCGGCAGACGGC-3′ were used with the reverse primer 5′-GTTTAAACTCACCGAATTGCAATCAGACC-3′ for STIP1 N-terminal deletion constructs. All PCR products were amplified at the following conditions: initial denaturation step at 95°C for 3 min, followed by 40 cycles of 95°C for 30 sec, 55°C for 30 sec, and 72°C for 90 sec plus a final extension (72°C for 10 min). The PCR products were double-digested with Asis I/Pme I (New England Biolabs, Ipswich, MA, USA) and ligated into the Asis I/Pme I-digested pFN21A vector (Promega). pNTAP-HSP90 and pNTAP-HSP90 ΔMEEVD were amplified from HA-HSP90 (Plasmid #22487, Addgene, Cambridge, MA, USA) using the forward primer 5′-GCGGATCCATGCCTGAGGAAGTGCACC-3′ and the following reverse primers: 5′-GATAAGCTTATCGACTTCTTCCATGCGAG-3′ (HSP90) and 5′-GATAAGCTTGCGAGACGCATCCTCATC-3′ (ΔMEEVD). The PCR conditions were as follows: initial denaturation step at 95°C for 3 min, followed by 40 cycles of 95°C for 30 sec, 50°C for 30 sec, and 72°C for 90 sec, plus a final extension (72°C for 10 min). PCR products were digested with BamHI/HindIII (New England Biolabs) and finally ligated into a BamHI/HindIII-treated pNTAP vector (Agilent Technologies, Santa Clara, CA, USA). pNTAP-JAK2, pNTAP-JAK2-840, and pNTAP-JAK2-251 were amplified from JAK2 cDNA (Sino Biological, Beijing, China) using the forward primer 5′-CTGCCCGGGCGGATCCATGGGAATGGCCTGCCTTAC-3′ with the following reverse primers: 5′-CGGTATCGATAAGCTTTCCAGCCATGTTATCCCTT-3′ (JAK2), 5′-CGGTATCGATAAGCTTATCCCGGTCTTCAAAGGCAC-3′ (JAK2-840), and 5′-CGGTATCGATAAGCTTTTTCAAGTTTCTGGCAGTGG-3′ (JAK2-251). PCR conditions were as follows: initial denaturation step at 98°C for 2 min, followed by 40 cycles of 98°C for 10 sec, 50°C for 30 sec, and 72°C for 60 sec, plus a final extension (72°C for 2 min) using the Q5 DNA polymerase (New England Biolabs). The PCR products were purified with spin columns and cloned with a BamHI/HindIII-treated pNTAP vector using an In-Fusion HD cloning kit (Clontech, Mountain View, CA, USA).

### DNA transfection and luciferase reporter assays

DNA transfection and luciferase reporter assays in ovarian cancer cell lines were performed as previously described in detail [14]. Briefly, cells were trypsinized and resuspended in serum-free RPMI medium at a concentration of 10^7^ cells/mL. Cell suspensions (200 μL) were mixed with 5 μg of reporter DNA and 20 ng of renilla plasmid. They were subsequently transferred to a 2-mm gap electroporation cuvette and pulsed at 120 Volts for 70 msec using a BTX ECM2001 pulse generator (BTX, San Diego, CA, USA). Cells were re-seeded into six-well plates and cultured in DMEM/F12 with 0.2% fetal bovine serum overnight. During the next day, cells were treated with IL-6 for 24 h, and luciferase activity was measured with a Dual luciferase reporter assay system (Promega) according to the manufacturer's instructions. STAT3, STAT5, and GAS1 (STAT1) reporters were purchased form Panomics (Affymetrix, Santa Clara, CA, USA). ARK2 and 293 cells were transfected using Lipofectamine 2000 (Invitrogen, Carlsbad, CA, USA) following the manufacturer's protocol.

### Transfection of small interfering (si)-RNA

MDAH2774 (3×10^6^ cells in 10-cm dish) were transfected with 50 nM of double-stranded RNA with the Lipofectamine RNAimax (Invitrogen) according to manufacturer's protocol. Small interfering-STIP1 and controls were purchased from Sigma Aldrich. After 72 h of transfection, suppression of the targeted genes was confirmed by RT-qPCR and western blot analyses.

### STIP1 monoclonal antibodies, peptide synthesis, and protein transfection

Antp-TPR peptide RQIKIWFQNRRMKWKKKA YARIGNSYFK [30] was purchased from GeneDireX (Las Vegas City, NE, USA). Peptide 520: (D-arginine) 8-EQKLISEEDLEHLKNPVIAQKIQKLMDVGLIAIR, scramble peptide: (D-arginine)8-EQKLISEEDLEKNHIVGILIKAQAIKVRPLQMLD were obtained from Kelowna International Scientific (Taipei county, Taiwan). All peptides were purified by high-pressure liquid chromatography (HPLC; > 80% purity), assessed by mass spectrometry, and then dissolved in PBS. Peptides and antibodies were transfected into ovarian cancer cells using the PULSin™ system (Polyplus, New York, NY, USA) according to the manufacturer's protocol.

### Western blot analysis

Cell lysates were prepared with RIPA buffer (150 mM NaCl, 20mM Tris-Cl pH7.5, 1% Triton X-100, 1% NP40, 0.1% SDS, 0.5% deoxycholate) containing freshly added proteinase and phosphatase inhibitors (Bionovas, Toronto, Canada). Protein concentrations were determined with the Bradford method. Each sample (50 μg) was subjected to electrophoresis in 10% SDS-polyacrylamide gels and then transferred onto nitrocellulose membranes. All antibodies were obtained from commercial sources, as follows: JAK1, JAK2, JAK3, and cleaved caspase 3 (Cell Signaling Technology, Danvers, MA, USA), TYK2, STAT3, and phospho-STAT3 Y705 (Abcam, Cambridge, UK), actin and STIP1 (Santa Cruz Biotechnology, Santa Cruz, CA, USA), and the corresponding horseradish peroxidase-conjugated antibodies (Santa Cruz Biotechnology). Enhanced chemiluminescence reagents were from Millipore Inc. The signal intensity of autoradiogram was quantified using the *UN-SCAN-IT* software (Silk Scientific, Orem, UT, USA). The relative intensity of each sample was normalized to the corresponding actin intensity.

### Dot blot and antibody neutralization assay

Three STIP1 monoclonal antibodies (H00010963-M02, MAB1626-M04, and MAB1626-M06) were obtained from Abnova (Abnova, Taipei City, Taiwan). Peptide 445−469 (STIP1 amino acids 445−469): TKAMDVYQKALDLDSSCKEAADGYQ, peptide 458−482 (STIP1 amino acids 458−482): DSSCKEAADGY QRCMMAQYNRHDSP, peptide 470−494 (STIP1 amino acids 470−494): RCMMAQYNRHDSPEDVKRRAMA DPE, peptide 482−506 (STIP1 amino acids 482−506): PEDVKRRAMADPEVQQIMSDPAMRL, peptide 495−519 (STIP1 amino acids 495−519): VQQIMSDPAMRLILEQMQKDPQALS, peptide 507−531 (STIP1 amino acids 507−531): ILEQMQKDPQALSEHLKNPVIAQKI and peptide 520−543 (STIP1 amino acids 520−543): EHLKNPVIAQKIQKLMDVGLIAIR were purchased form GeneDireX (Las Vegas City, NE, USA). All of the peptides were dissolved in PBS and then spotted on a 0.2 m PVDF membrane (Bio-Rad, Hercules, CA, USA) at different concentrations. After air drying, the membrane was treated using the same protocol utilized for western blot. Peptide detection was performed with a STIP1 monoclonal antibody. The antibody neutralization assay was performed using a STIP1 monoclonal antibody. Peptides at different molar ratios were pre-incubated at room temperature for 2 h. The mixtures were probed with a membrane spotted with rhSTIP1 [6] at different concentrations. RhSTIP1 was detected with the methodology used for western blotting.

### RNA extraction and real-time qPCR

Total RNA was isolated with the TRIzol reagent (Invitrogen). First-strand cDNA for RT-qPCR was synthesized with an oligo-T primer using superscript III first strand synthesis kit (Invitrogen). The expression patterns of STIP1, JAK2, and GAPDH mRNAs were analyzed with the TaqMan^®^ GENE expression assay (Applied Biosystem).

### Immunohistochemistry

FFPE ovarian cancer tissues were sectioned at 4 μm thickness and deparaffinized with xylene. After rehydration in a series of graded ethanol solutions, sections were stained with anti-human STIP1 (Abnova, Taipei City, Taiwan) and anti-JAK2 (Cell Signaling Technology, Danvers, MA, USA) antibodies in an automated stainer for immunohistochemistry (Leica Bond Polymer Refine Detection Kit; Buffalo Grove, IL, USA) according to the manufacturer's protocol. Hematoxylin was used for counterstaining. A semiquantitative assessment of immunohistochemical staining was performed using a histoscore calculated by multiplying the percentage of tumor cells (0−100%) by the immunointensity (0−3) as previously described [45].

### Proximity ligation assay

Cell lines were seeded onto chamber slides for 24 h. The protocol for immunofluorescence microscopy was used for cell fixation. Deparaffinization of paraffin-embedded ovarian cancer sections was performed with the same protocol used for immunohistochemistry. After incubation in blocking solution (Thermo scientific, Walthma, MA, USA) for 1 h, slides were stained with a combination of JAK2, HSP90 (Cell Signaling Technology), STAT3 (Abcam), STIP1 (Abnova) as well as an IgG control antibody (Sigma). The interactions between proteins were examined with a recently developed commercial assay (Duolink *in situ* Red starter kit mouse/rabbit) based on the principle of proximity ligation. The slides were then examined using a Leica TCS SP2 laser scanning confocal system (Leica Microsystems GmbH, Wetzlar, Germany).

### Cell viability assay

MDAH2774, SKOV3, and ARK2 cells were plated in 96-well culture plates at 6000 cells per well. Cells were treated with the reported concentrations of scramble peptide or peptide 520 in serum-free DMEM/F12 for 24 h before being used in the assays. The effect of peptide 520 on cell viability was measured with the MTT (3-(4,5-dimethylthiazol-2-yl)-2,5-diphenyltetrazolium bromide) assay (Sigma). The optical density was measured at a wavelength of 570 nm using an automated scanning spectrophotometer (Wallac Victor2 spectrophotometer; Perkin-Elmer, Boston, MA, USA).

### Immunofluorescence microscopy

Cells were cultured overnight on cover slides at the concentration of 3×10^5^ cell per well in a 6-well plate and then exposed to serum starvation conditions for additional 24 h. After incubation with scramble peptide or peptide 520 (1 μM) for 24 h at 37°C, cells were fixed with 2% paraformaldehyde at 4°C for 30 min and then incubated for 1 h at room temperature in blocking buffer (5% normal goat serum in PBS) to reduce non-specific binding. Myc staining was performed by incubating cells with a mouse monoclonal anti-myc antibody (Invitrogen, 1:100) for identifying synthetic peptides. After incubation with anti-Alexa Fluor-488 (1:100, Invitrogen), slides were mounted with the Vectashield mounting medium (Vector Laboratories) and analyzed using a Leica TCS SP2 laser scanning confocal system (Leica Microsystems GmbH).

### Immunoprecipitation

Cell lysates were prepared with cell lysis buffer (20 mM Tris-Cl pH7.4, 25 mM NaCl and 0.1% NP40) containing proteinase inhibitors. Proteins (2 mg) were incubated with control IgG, monoclonal anti-STIP1antibody (Abnova) or anti-HSP90 antibody (Abcam) at 4°C overnight under agitation. Enriched protein complexes were concentrated with protein A agarose beads (Millipore, Billerica, MA, USA). Agarose beads were washed for three times with wash buffer (20mM Tris-Cl pH7.4, 25mM NaCl) and boiled with sample buffer (0.25 M Tris-Cl pH 6.8, 0.08% SDS, 20% glycerol, and 10% β-mercaptoethanol). Immunoprecipitation was performed with an ImmunoCruz IP/WB optima system (Santa Cruz Biotechnology). Each sample was subjected to electrophoresis with 8% SDS-PAGE. Antibodies raised against JAK2, HSP90 (Cell Signaling Technology), STAT3 (Abcam), and STIP1 (Abnova) were used for western blot analysis. Halo fusion proteins, NTAP proteins, and His-tagged ubiquitin were pulled down using the Halo-tag protein purification system sample pack (Promega), streptavidin agarose (Sigma) and nickel beads (Invitrogen), respectively, following the manufacturer's protocols.

### Animal studies

All animal procedures were performed in accordance with the institutional review board-approved protocol (IACUC No. 2013121103). MDAH2774 cells (1×10^6^ cells) suspended in Hank's balanced salt solution (HBSS; 100 μL) were subcutaneously injected into nude mice using a 23-gauge needle (Becton Dickson, Franklin Lakes, NJ, USA). When the tumor reached 10 mm^3^ in volume, the mice were divided into two groups (vehicle and peptide 520). Vehicle (PBS) or peptide 520 (> 95% purity, 25 μg/50 μL) were directly injected into the tumor three times per week. Tumor volume was calculated as follows: width × length × height (in mm).

## SUPPLEMENTARY MATERIALS FIGURES


