# Supplementary material for: Stress-induced phosphoprotein-1 maintains the stability of JAK2 in cancer cells
Source: Oncotarget. 2016 Jul 8;7(31):50548–63. doi: 10.18632/oncotarget.10500 (PMC5226602; doi:10.18632/oncotarget.10500)
Supplement: Supplementary file 1 [file oncotarget-07-50548-s001.pdf]

Stress-induced phosphoprotein-1 maintains the stability of JAK2 in cancer cells

SUPPLEMENTARY FIGURES

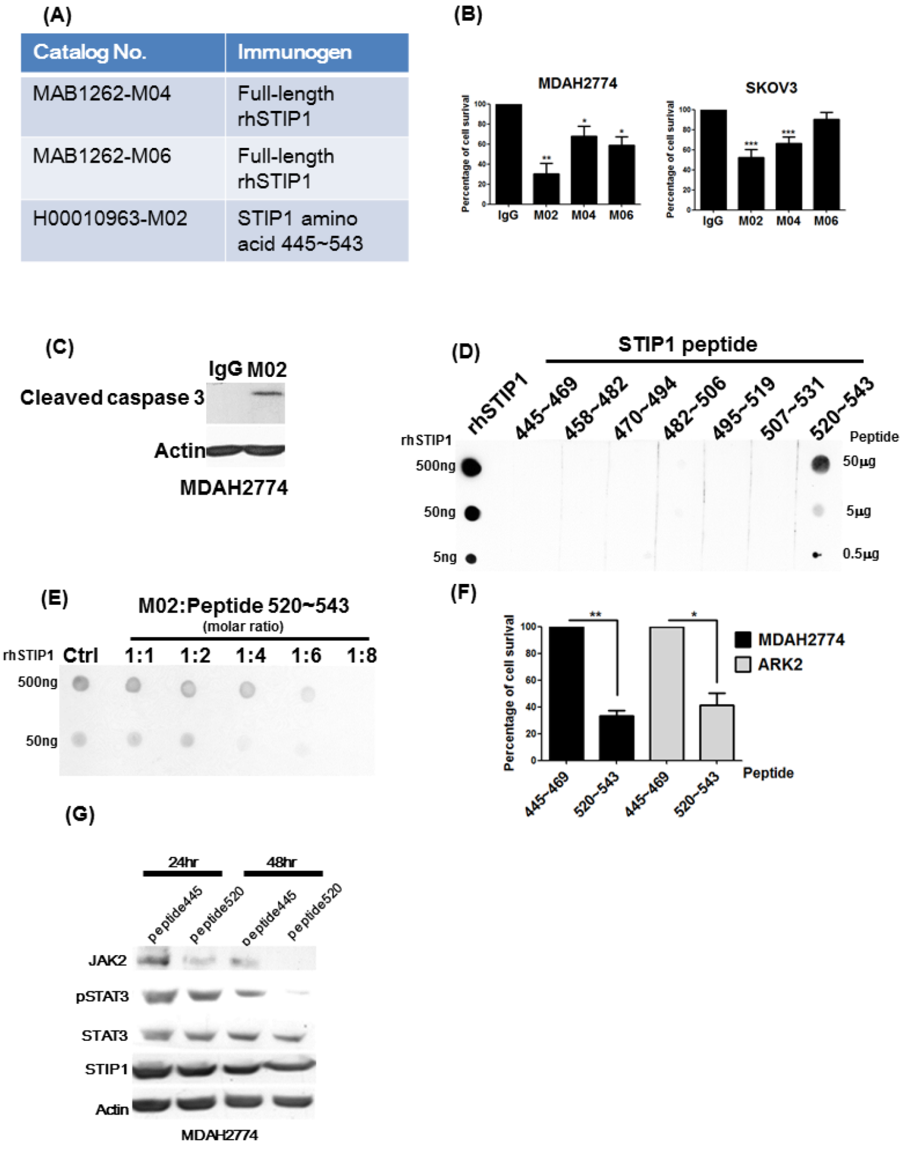

Supplementary Figure S1: STIP1 monoclonal antibody and its corresponding immunogenic peptide induced cancer cell death. A. Three different STIP1 monoclonal antibodies induced by STIP1 immunogens. **B, C.** MDAH2774 and SKOV3 cells were transfected with M02, M04, and M06 antibody. Cell survival was measured by MTT assay (B). Protein levels of caspase 3 activation were measured by western blot (C). **D.** Epitope mapping of M02 antibody. Seven synthetic peptides generated from STIP1 (amino acid sequence between 445 and 543) were dotted onto the membrane and hybridized with M02 antibody. rhSTIP1 was a positive control for the dot blot. **E.** After pre-incubation with M02 antibody, Peptide 520 (amino acids 520~543) neutralized M02 antibody for the detection of rhSTIP1 at different molar ratio. **F.** MDAH2774 and ARK2 cells were transfected with 20μM Peptide 520 or Peptide 445~469 that does not include DP2 domain for 24 h. Cell survival was measured by MTT assay. **G.** MDAH2774 cells were transfected with 20μM peptide 445 or peptide 520 for 24 and 48 h and analyzed with western blot using indicated antibodies.

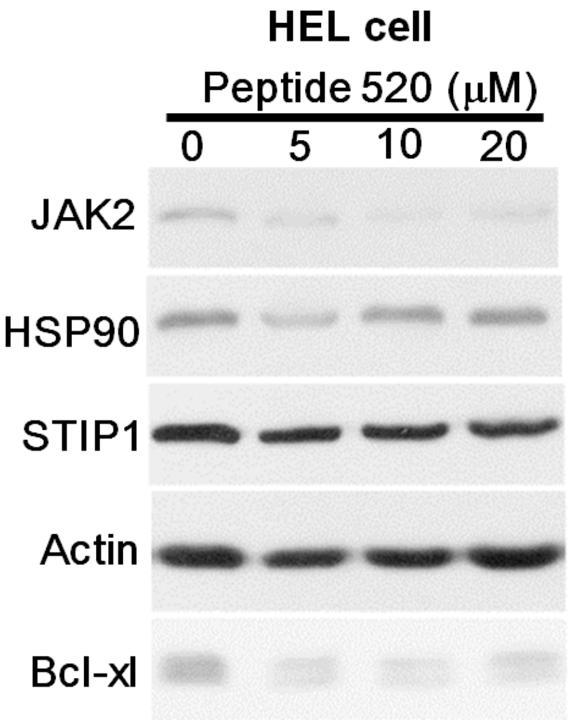

Supplementary Figure S2: Peptide 520 represses JAK2 expression in HEL cells. (JAK2-mutant cells at V617F). HEL cells were treated with Peptide 520 in serum free medium for 48 h. Protein levels of JAK2, HSP90, and Bcl-xl were detected by western blot.

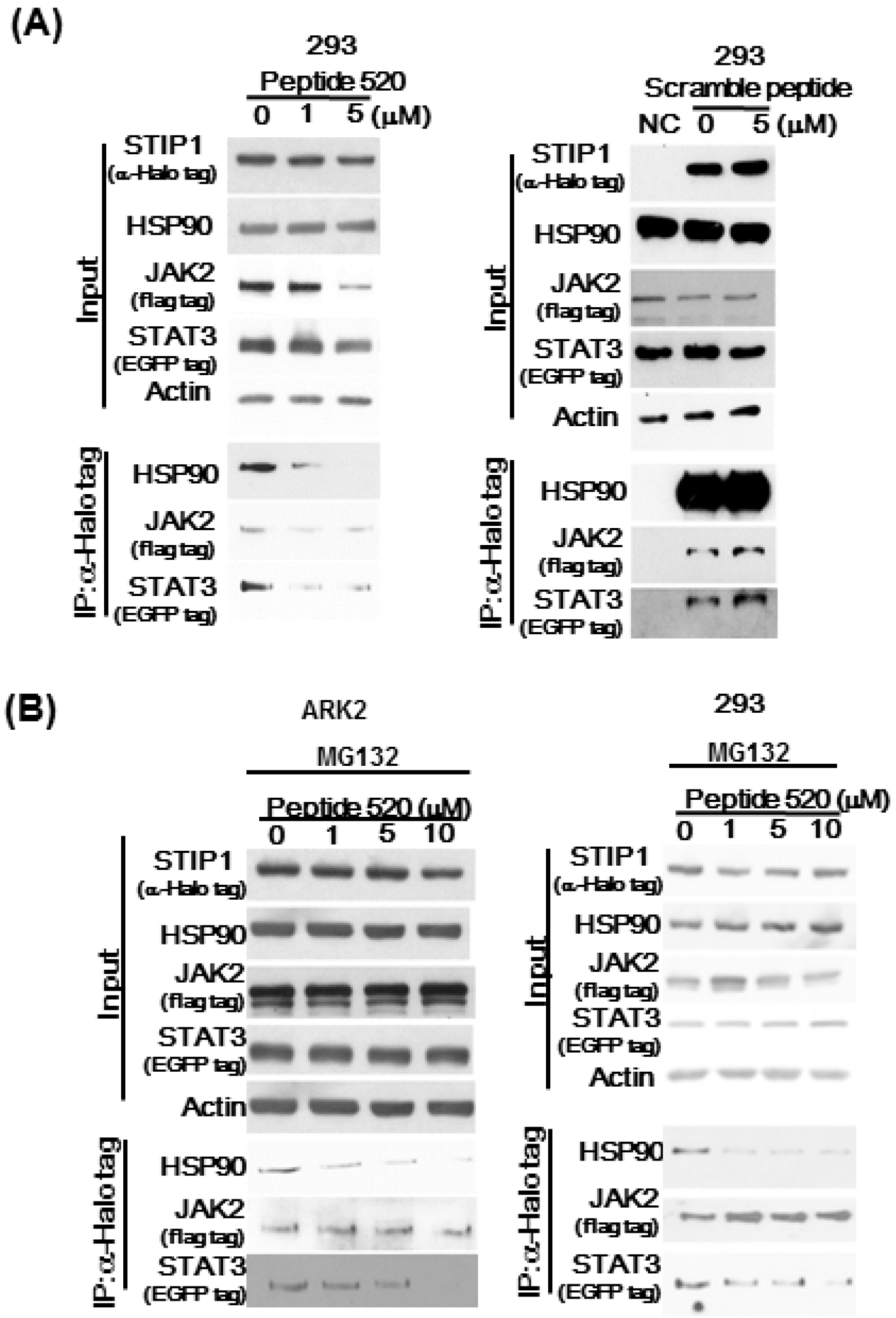

Supplementary Figure S3: Peptide 520 inhibited STIP1-JAK2 binding to HSP90 and STAT3 in a dose-dependent manner. ARK2 and 293 cells were transfected with different concentrations of peptide 520 (A, left panel) or scramble peptide (A, right panel), Halo-STIP1, Flag-JAK2, and EGFP-STAT3, followed by being pulled-down with Halo-tag resin in the absence **A.** or presence **B.** of MG132. In the NC (negative control) experiments, expression vector for Halo-STIP1 was not used. Protein levels of JAK2 and STAT3 were detected with western blot.

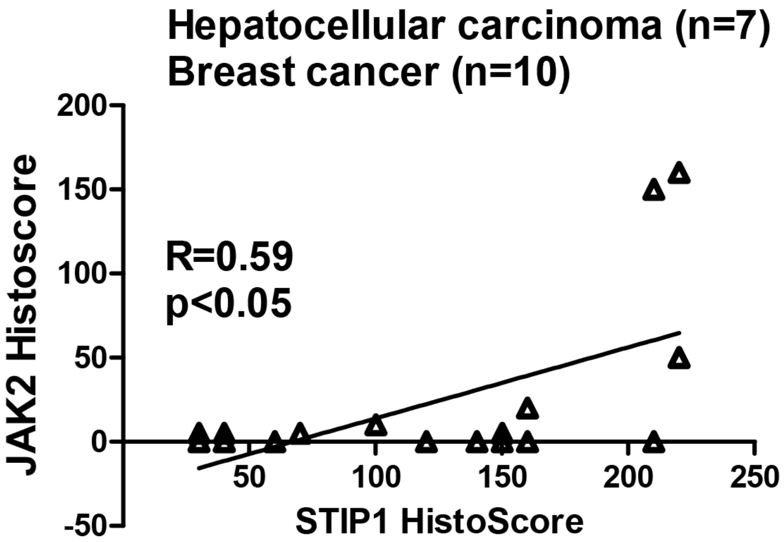

Supplementary Figure S4: STIP1 and JAK2 are co-expressed in cancer tissues. Correlation of STIP1 and JAK2 histoscores between STIP1 and JAK2 is examined in other carcinomas, i.e. hepatocellular carcinoma (n = 7) and breast cancer (n = 10). The overall R value is 0.59 and p <0.05.
